# Distribution and viability of ocular and non-ocular *Chlamydia trachomatis* in households in a trachoma-endemic community in Oromia, Ethiopia

**DOI:** 10.1371/journal.pntd.0012759

**Published:** 2025-01-13

**Authors:** Oumer Shafi Abdurahman, Gebeyehu Bekele, Robert Butcher, Gadissa Deressa, Asanti Mumme, Munira Mohammed, Rufia Nure, Kedir Temam Nuri, Gemeda Shuka, Korso Hirpo, Katie Greenland, Esmael Habtamu, Bart Versteeg, David Macleod, Anna Last, Matthew J. Burton

**Affiliations:** 1 International Centre for Eye Health, Clinical Research Department, London School of Hygiene & Tropical Medicine, London, United Kingdom; 2 The Fred Hollows Foundation, Addis Ababa, Ethiopia; 3 Adama Regional Referral Laboratory, Adama, Ethiopia; 4 Department for Disease Control, London School of Hygiene & Tropical Medicine (LSHTM), London, United Kingdom; 5 Knowledge Institute of the Dutch Association of Medical Specialists, Utrecht, the Netherlands; 6 Department of Infectious Disease Epidemiology, London School of Hygiene & Tropical Medicine, London, United Kingdom; 7 National Institute for Health Research Biomedical Research Centre for Ophthalmology at Moorfields Eye Hospital NHS Foundation Trust and UCL Institute of Ophthalmology, London, United Kingdom; RTI International, UNITED REPUBLIC OF TANZANIA

## Abstract

**Background:**

We aimed to determine the household distribution and viability of *Chlamydia trachomatis (Ct)* from the eyes, face, and hands during the initial two visits of a year-long fortnightly cohort study in geographically defined adjacent households.

**Methods/Findings:**

We enrolled 298 individuals from 68 neighbouring households in Shashemene Woreda, Oromia, Ethiopia. All individuals above 2 years of age residing in these households were examined for signs of trachoma. Swab samples were taken from the conjunctiva, faces, and hands and analysed for the presence and viability of *Ct*. *Ct* viability was determined using reverse transcription (RT) PCR.

At the initial visit, out of 298 individuals, 133 (44.5%) were children aged 2–9 years. Among these children, 27/133 (20.3%) had trachomatous inflammation—follicular (TF), while 8/133 (6.0%) had trachomatous inflammation—intense (TI). *Ct* (omcB or pORF2) was detected in 16/133 (12.0%) eye swabs, 14/105 (13.5%) face swabs, and 11/105 (10.5%) hand swabs from children aged 2–9 years. Among these children at visit one, 12/14 (85.7%) with *Ct* on faces and 9/11 (81.8%) with *Ct* on hands also had detectable ocular *Ct*. The severity of the disease worsened from the first visit to the second, and no participants showed clearance of the disease within the two-week period. *Ct* infection was associated with TF (P = 0.002) and TI (P = 0.060).

At visit one, among children aged 2–9 years, viable *Ct* was detected in 12/16 (75.0%) ocular, 6/14 (42.9%) face, and 4/11 (36.4%) hand swab samples. All viable *Ct* detected on the faces and hands were identified from individuals with viable ocular infections. Among caregivers whose child tested positive for Ct on their hands, 3 caregivers also had Ct on their hands, accounting for 20% (3 out of 15). Additionally, among caregivers whose child tested positive for Ct on their faces, 2 caregivers had Ct on their faces, which accounts for 14.3% (2 out of 14). In two participants, we detected Ct on the hands of ocular-negative children at the initial visit and later detected ocular Ct at the second visit.

**Conclusion/Significance:**

Using RT-qPCR assay to detect *Ct* omp2 mRNA to define viability offers a new, informative perspective of trachoma transmission in this community in Ethiopia. The presence of viable *Ct* on the faces and hands of individuals living in households with people with current ocular *Ct* infection supports the hypothesis that hands and faces are important routes for transmission of trachoma. This highlights the importance of targeted interventions to address these sites of *Ct* carriage to help interrupt transmission.

## Author summary

Trachoma, caused by *Chlamydia trachomatis* (Ct), remains a leading cause of infectious blindness globally. Understanding how Ct is transmitted within households is crucial for designing effective interventions. In this study, we investigated the presence and viability of Ct on the eyes, faces, and hands of individuals in geographically adjacent households in Shashemene Woreda, Oromia, Ethiopia, over two visits during a year-long cohort study.

We enrolled 298 participants from 68 neighbouring households, including 133 children aged 2–9 years. Trachomatous inflammation—follicular (TF) was observed in 20.3% of these children, and trachomatous inflammation—intense (TI) was found in 6.0%. Ct was detected in ocular, facial, and hand swabs, with a strong association between ocular Ct infection and the presence of viable Ct on the face and hands. Notably, 75.0% of ocular Ct infections in children were viable, as were 42.9% and 36.4% of infections on the face and hands, respectively. Viable Ct on the face and hands was predominantly identified in individuals who also had viable ocular infections.

Caregivers of children with Ct on their faces or hands occasionally carried Ct themselves, underscoring potential intra-household transmission. We also observed cases where Ct on the hands preceded ocular infection, supporting the hypothesis that hands and faces play a critical role in disease spread.

By utilizing reverse transcription PCR to detect viable Ct, this study provides an insights into transmission dynamics in this Ethiopian community. The findings emphasize the need for targeted interventions focusing on facial and hand hygiene to disrupt Ct transmission and support ongoing trachoma elimination efforts.

## Introduction

Trachoma is caused by ocular infection with *Chlamydia trachomatis (Ct)* and is the leading infectious cause of blindness globally [1]. The pathogenesis of trachoma begins in early childhood and can progress through the life course. Children living in endemic communities experience repeated episodes of *Ct* infection which provokes conjunctival inflammation, usually followed by resolution. This repeated cycle of chronic inflammation can result in conjunctival scarring of the inner surface of the eyelids, which, if severe, distorts the eyelid and brings the eyelashes into contact with the eye (trichiasis). Abrasive damage from trichiasis causes corneal opacification and vision impairment [2].

The World Health Organization (WHO) has targeted the worldwide elimination of trachoma as a public health problem by 2030. One of the criteria for elimination is the reduction of the prevalence of trachomatous inflammation—follicular (TF) in 1−9-year-olds (TF_1-9_) to <5% in all endemic districts, using The WHO simplified grading system recommended for programs [3]. In research settings, the modified WHO grading system describes a detailed grading of the upper tarsals. In the modified WHO system, the upper tarsus is divided into three zones, with upper tarsal follicles (F) graded from F0 to F3 based on their quantity: **F0**: no follicles, **F1**: 1–5 follicles in zones 2 and 3, **F2**: more than 5 follicles in zones 2 and 3 but fewer than 5 in zone 3, **F3**: 5 or more follicles. F2 and F3 are comparable to TF in the WHO simplified system.

Upper tarsal papillary hypertrophy and diffuse infiltration (P) are graded as follows: **P0**: normal appearance, **P1**: individual vascular tufts prominent but deep vessels are visible, **P2**: more prominent papillae, with hazy vessels, **P3**: pronounced conjunctiva, thickened and opaque, hiding vessels over more than half the surface.

P3 corresponds to TI in the WHO simplified system [4].

The WHO recommends the use of the SAFE strategy to control trachoma. This involves **S**urgery for trichiasis, **A**ntibiotic treatment, promotion of **F**acial cleanliness and **E**nvironmental improvements. The antibiotic component is mostly delivered as annual rounds of mass drug administration (MDA) with azithromycin, to reduce the community burden of infection. The F and E components are intended to reduce the transmission intensity of *Ct*.

As of April 2023, WHO estimates that 115.7 million people need to receive implementation of the SAFE strategy. 55% of the global population living in districts in need of interventions against trachoma live in Ethiopia [5]. Despite the many years of continuous implementation of the standard SAFE strategy in some regions of Ethiopia (particularly the Amhara and Southern Nations, Nationalities and Peoples region), trachoma elimination has not yet been achieved [6,7]. To achieve the 2030 elimination goals, national trachoma control programmes need more effective disease control approaches, particularly to reducing *Ct* transmission. Research is needed to better understand the potential routes of ocular Ct transmission and their relative importance to better define opportunities for intervention.

Recently, we reported a study mapping potential *Ct* transmission routes in a trachoma-endemic village in Oromia Region, Ethiopia. We tested swabs from ocular and non-ocular surfaces (hands, faces and fomites) and flies caught leaving the faces of children for *Ct* using a quantitative PCR (qPCR) assay. We demonstrated that in households with current cases of ocular *Ct* infection, *Ct* DNA could be detected in some samples from faces, hands, clothes, and flies [8].

In common with our study described above, most diagnostic tests for *Ct* used in trachoma research detect chlamydial DNA or long-lived RNA targets. These targets can persist long after the pathogen has become non-viable [9]. Viable pathogens are required for transmission, therefore identifying which of the DNA results we find come from viable pathogens could help clarify transmission routes, not discernible from DNA data alone. The gold standard method to detect viable *Ct* is cell culture because of its high specificity. However, *Ct* culture is low throughput and sensitivity is low. Viability can also be inferred from the propidium monoazide (PMA) exclusion method, where PMA treatment of the diagnostic sample crosslinks DNA of pathogens without an intact outer membrane and blocks polymerase-based amplification [10,11]. However, a limitation of the PMA exclusion method is that some non-viable bacteria may not lose membrane integrity and the proportion of truly viable bacteria may be overestimated [10,12,13]. Detection of *Ct*-specific RNA is presumed to indicate an infection is metabolically active [14], and is increasingly being used as a marker of viability, particularly in the context of an urogenital Ct infection [15–17]. Detection of rRNA has been used as a marker of viability of infection in the context of ocular Ct infections, and the diagnostic advantages of using a high copy number rRNA target have been described [18,19]. However, rRNA can be detected 3–4 weeks after an infection has been treated, which makes it unsuitable for an intensive (two weekly) follow up of natural infection history. Conversely, many of the most highly expressed mRNA transcripts, including outer membrane protein transcripts, have a half-life of ~15 minutes, suggesting they degrade quickly once a chlamydial body ceases transcription [20]. We therefore opted to use mRNA detection as a marker of infection viability in this study.

We have previously investigated the *in vitro* durability of detectable *Ct* (both viable and non-viable) on potential fomite surfaces (spiked with different concentrations of *Ct*), such as pig skin, cloth, and plastic [10]. This found that *Ct* DNA remained detectable and stable for 24 hours. In contrast, the amount of viable *Ct*, assessed by a PMA-based viability assay, declined sharply over the same period. Here, we assess the viability of Ct DNA found on faces and fingers in a field setting.

Here, we describe two consecutive visits to one community, two weeks apart, which establish the suitability of this community for further longitudinal follow-up. This study describes the household distribution and the viability of ocular and non-ocular *Ct* and trachomatous disease in all households in a defined geographical area with a view to identifying potential routes of transmission for further study.

## Methods

### Ethical approval

This study was approved by the National Research Ethics Review Committee of the Federal Ministry of Science and Higher Education of Ethiopia (Ref: MoSHE/RD/14/1/8824/20) and the London School of Hygiene & Tropical Medicine Ethics Committee (LSHTM Ethics Ref: 15928). Written informed consent was obtained from all individuals 18 years and older. For individuals younger than 18 years, parents/guardians provided written informed consent, and additionally written assent was obtained from those aged 7 to 17 years.

### Study design

A 24 time point cohort study was initiated, during which participants were followed every two weeks from February 19, 2020 (at the end of the dry season, known as Bega). The cohort study was interrupted on March 24, 2020 (the start of the short rain season, also known as Belg) due to the Covid-19 pandemic, during which time all community-based public health activities were suspended. In this paper, we describe the initial two consecutive visits to the cohort community as a baseline for the cohort. The subsequent follow-up visits resumed from October 2020 and persisted through October 2021, encompassing 22 visits with the same community. Data from these 22 visits will be reported later.

### Study site and population

This study was conducted in Shashemene woreda of Oromia region, Ethiopia. Before the study began, nine sub-kebeles (zones) from three adjacent kebeles in Shashemane woreda were screened by trained and validated ophthalmic nurses who assessed a convenience sample of 100 children who presented for examination in each village centre for clinical signs of trachoma based on the WHO trachoma grading guideline [21]. The study village, 14 kilometres from Shashemene town, was purposively selected as it had the highest TF prevalence (30%) among children aged 1–9 of the sub-kebeles screened. Within the sub-kebele, we selected 68 consecutive households that met the inclusion criteria, starting from the end of a block and proceeding until we reached the 68th household. The first (baseline) visit occurred three weeks after completing the assessment and census of the selected households.

To be eligible for inclusion in this cohort study, households needed to have one or more children aged 2–9 years at the time of enrolment and everyone aged 2 years and above was included. This age range was applied because of the high follow-up frequency (every two weeks for one year). Prior to the start of the study, the community received azithromycin Mass Drug Administration (MDA) from the national trachoma control program in July 2016, December 2017, and July 2018. However, MDA was not carried out in 2019 due to security concerns, and not carried out in 2020 because of the COVID-19 pandemic.

### Sample size

A cohort of 68 households was enrolled in a one-year longitudinal study, with fortnightly data collection visits. Sample size for the cohort was determined to estimate the mean duration of infection/disease in line with the overall aims of the cohort study. To estimate with a margin of error of +/- 5 weeks, and assuming a standard deviation of 20 weeks [22], we would need to observe 62 episodes of infection/disease. We assumed an average of two infections per household over the course of the year, and a loss to follow-up of 10%, which would necessitate recruiting 34 households. Given the geographical sampling of adjacent households, the clustered nature of trachoma and the possibility of fewer than two infections per household, we inflated this to recruit 68 households to the cohort, which was feasible within the fortnightly visit schedule. New children and residents are included to the cohort if they either turn two years old or are identified after the baseline visit.

### Household survey

At the first visit, the socio-demographic and household characteristics, as well as potentially associated risk factors for trachoma, were assessed using a pre-piloted questionnaire. This questionnaire had been used in our prior study within the same woreda [23]. The questionnaire was administered to each primary caregiver and the gathered information was entered into an electronic device using Open data kit (ODK) software system. Data collectors observed latrine availability and determined utilization by looking into and around the latrine. Additionally, the GPS location of the 68 households surveyed was recorded during the study. To investigate potential contact between children, which may facilitate the transmission of *Ct*, we documented family relationships within the community.

### Clinical examination, ocular and non-ocular sample collection

A single ophthalmic nurse was standardised for clinical conjunctival examination using the WHO simplified trachoma grading system [24] against an expert grader in a village separate from the study community. Grader standardisation was conducted on clinical cases rather than conjunctival photographs and achieved a weighted kappa score of >0.9 for F and >0.7 for P compared to a highly experienced grader before their deployment to the field. On each study visit, all consenting, available and eligible participants were examined by a validated ophthalmic nurse. Presence of ocular or nasal discharge and any fly-face contacts (during the period of examination) were documented. Ocular discharge was defined as “the presence of clear or cloudy fluid or dry matter on the eyelid margin or eyelid (including the corners) and nasal discharge as the presence of wet or dry discharge visible outside the nares” [25]. At both time points, examinations were conducted between 8 and 11 am. Both eyes were examined for trachoma by the ophthalmic nurse using the WHO simplified [4] and modified WHO FPC grading systems using a 2.5X binocular loupe with adequate light. High-resolution photographs were taken of the left eye of every participant using a digital SLR Nikon D7200 camera body with a 105mm macro lens.

A designated trained photographer took all the photographs. The same photographer and ophthalmic nurse team worked together throughout the study. To ensure data quality, in addition to the field grading, the conjunctival photographs were independently graded by a single experienced ophthalmologist. The photographs were graded using the modified FPC system [24]. The ophthalmologist’s photographic grades were used in this analysis.

One conjunctival swab sample was collected from the left upper tarsal conjunctiva of each participant using the following procedure. A sterile polyester-tipped swab (Puritan) was wiped four times across the everted tarsal conjunctival surface, rotating the head of the swab by a quarter turn with each sweep. Swabs were also collected from the hands and faces of all individuals. For the face and hand swabs, each swab was first moistened with distilled water. One swab was used to trace a line under the left eye, across the cheek, and under the left nostril. A separate swab was used to collect samples from the hands, rubbing across the palms and the backs of both hands [25]. At visit one, the conjunctival, hand and face swabs were immediately placed separately in a tube containing 300μL of RNALater to stabilise the RNA to allow for viability testing. At visit two, only conjunctival swabs were put in RNALater due to resource restrictions. Hand and face swabs were collected in distilled water. Control swabs were selected at random from the swab dispenser box and were performed using the same no touch technique and moving the swab in the air for 20 seconds in front of the surface but without touching the surface (eyes, face or hands). All samples were placed immediately in an insulated box with ice packs in the field and then transferred to a -20°C freezer for storage later the same day, before onward transfer using a cool box and storage at -80°C within two weeks. The processing and analysis of the samples were carried out in Ethiopia at the Oromia Regional Laboratory in Adama.

### DNA and RNA extraction and detection

DNA and RNA were extracted from swabs using a RNA/DNA purification kit (Norgen), with on-column RNAse-free DNAse-1 treatment (Norgen) of the RNA. Both extraction and RNAse-free DNAse kits were used according to the manufacturers’ guidelines. DNA was eluted in 100 μL and RNA was eluted in 50 μL.

DNA was detected using a qPCR assay targeting two *Ct* targets (outer membrane complex B [omcB], a single-copy quantitative target, and plasmid Open Reading Frame 2 [pORF2], a multicopy qualitative target to maximise sensitivity) and one human target (RNAse P subunit [RPP30]), serving as an endogenous sample adequacy and processing control [26]. 20-μL assays were prepared containing 4-μL template DNA and TaqMan Multiplex Master Mix at 1X concentration (ThermoFisher Scientific). Oligonucleotide concentrations were optimised for our platform. The pORF2 and RPP30 probes were included at 0.5 μM and 0.1 μM, respectively, in the final reaction. The other oligonucleotides were included at 0.3 μM in the final reaction. A six-step, ten-fold dilution series of known concentration standards and no-template PCR controls were run on each plate.

The quantitation cycle (Cq) range for baseline fluorescence estimation and fluorescence thresholds were set manually and standardised across plates. Samples were considered valid if (1) there was linear correlation between plate standard concentration and Cq value, (2) the plate no-template controls were negative, and (3) the endogenous control RPP30 target amplified within 40 cycles. Samples were considered positive for *Ct* if either omcB or pORF2 targets amplified within 40 cycles. Sample load was determined by extrapolating copies/μL of omcB target from the Cq value (positive samples where no omcB amplified were not quantified).

Pathogen viability was examined in *Ct* DNA-positive samples. Viability was tested using reverse transcription (RT) PCR. 8 μL of RNA from each sample was treated with ezDNAse (ThermoFisher Scientific) according to the manufacturer’s guidelines. DNA-free RNA was then reverse transcribed using the SuperScript III first-strand synthesis system (ThermoFisher Scientific) according to manufacturer’s guidelines. 8 μL of DNA-free RNA was primed using gene-specific primers for the constitutively expressed *Ct* gene outer membrane protein 2 (omp2) [27]. The primed RNA was incubated with SuperScript III reverse transcriptase to create first strand complementary DNA (cDNA), and then the template RNA was removed with RNAse H incubation. cDNA was then amplified in 10 μL-μL PCR reactions using previously published omp2 primers at 0.2 μM and probe at 0.1 μM, TaqMan Multiplex Master Mix at 1X and 2 μLof cDNA template. DNAse-treated RNA which had not been subjected to RT from each sample was also tested using the same assay as a no-RT control. Samples were run on plates with serial dilutions of known-concentration standards and no-template controls. Samples were considered valid if the plate controls performed as expected and there was no amplification in the paired no-RT controls. Samples were considered viable if omp2 cDNA amplified with 40 cycles.

### Statistical analysis and geographical mapping

Data were analysed using Stata (StataCorp. 2019. Stata Statistical Software: Release 16. College Station, TX: StataCorp LLC). Disease and laboratory infection data at both time points with the baseline GPS locations of each household were checked for correctness of household and personal identification numbers, age, any duplicate etc. and merged using Stata. Household mapping was performed using QGIS 10.7.1 and visualised on an OpenStreetMap base map.

*Ct* positivity was defined as any ocular swab positive for either omcB or pORF2. Univariable logistic regression analysis was utilized to explore the association between *Ct* infection and clinical trachoma (TF, TI and detailed clinical grading) for children aged 2–9 years old for each visit, where the cell numbers are less than five, we should use Fishers exact test. An individuals’ Ct load was categorised as high or low, based on whether the number of DNA copies per uL was higher or lower than the geometric mean of Ct load in children aged 2–9 years across the two timepoints (29 copies/uL) [23].

The association between *omcB* load category and active trachoma, defined as presence of TF and/or TI, was tested using the Chi-square (χ^2^) Fisher exact test. Additionally, the χ^2^ test was used to compare variables between visit 1 and visit 2 to assess any significant differences over time. Household Intra-cluster correlation (ICC) was reported based on cases clustering within households using a random effects probit model with *Ct* infection/TF/TI (in turn) as the outcomes, which allows for differing household sizes. ICC above 0.5 was considered a level high clustering. Finally, a detailed analysis of viable *Ct* was described.

## Results

### Study households and participants

During the first visit, there were 298 participants present, and 257 were present at the second visit. At the initial visit, there were 165 females (55.4%), and 133 (44.6%) were aged between 2 and 9 years. Fewer participants were present at visit two, however, they had similar demographic characteristics to the people seen during visit one. Individual demographic / clinical characteristics and general household characteristics are presented in Table 1.

**Table 1:** Socio-demographic and clinical characteristics of the study community at first visit.

Categories		Visit round			
		Visit one		Visit two	
		n/N	%	n/N	%
**Individual characteristics**					
Sex	Male	134/298	45	103/257	40.1
	Female	165/298	55	154/257	59.9
Age (years)	2–9	133/298	45	126/257*	49
	10+	166/298	56	131/257	51
	Median	12		10.8	
	IQR	6–25		6–25	
TF	Age 2–9	27/133	20	37/126	29.4
	All ages	27/298	9	39/256	15.2
TI	Age 2–9	8/133	6	15/126	11.9
	All ages	8/298	2.7	18/257	7
Ocular discharge	Age 2–9	24/133	18	7/126	5.6
	All ages	28/298	9.4	8/257	3.1
Nasal Discharge	Age 2–9	38/133	29	25/126	19.8
	All ages	38/298	13	25/257	9.7
Flies on face	Age 2–9	50/133	38	27/126	21.4
	All ages	62/298	21	29/257	11.3
**Household characteristics**					
Latrine	Available (utilized)^+^	34/68	50		
	Available (not utilized)	3/68	4.4		
	Not available	31/68	46		
Water source during dry season (MV* = 1)	Borehole/standpipe	36/68	52		
	Surface water	31/68	46		
Number of rooms used for sleeping (MV* = 1)	One	33/68	49		
	Two	32/68	47		
	Three	3/68	4.4		
Household head education level (MV* = 5)	No education	5/63	7.9		
	Some primary	17/63	27		
	Completed primary	25/63	40		
	Some secondary	8/63	13		
	Completed secondary	3/63	4.8		
	Higher education	5/63	7.9		

*MV = missing value

Abbreviation: IQR–Inter quartile range.

^+^ The data collector records latrine utilization upon observing signs indicating its usage.

The difference in number of children visited was 7 in number. However, 13 children who were visited at visit one were missed at visit two while 8 children who were absent from visit 1 were visited at visit two. 113 children were visited at both time points.

**Fig 1:**
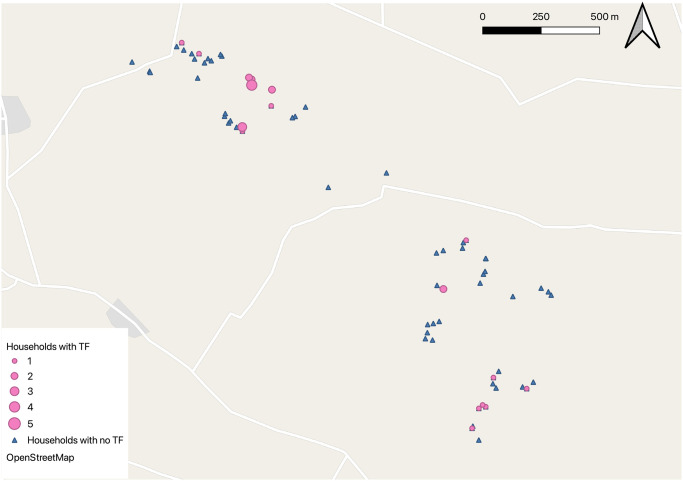
Map of the study area showing a scattered distribution of Trachomatous Inflammation-Follicular (TF) at visit one. Geographical maps were created using QGIS and Map base layers taken from OpenStreetMap https://www.openstreetmap.org/copyright generated in QGIS.

**Fig 2:**
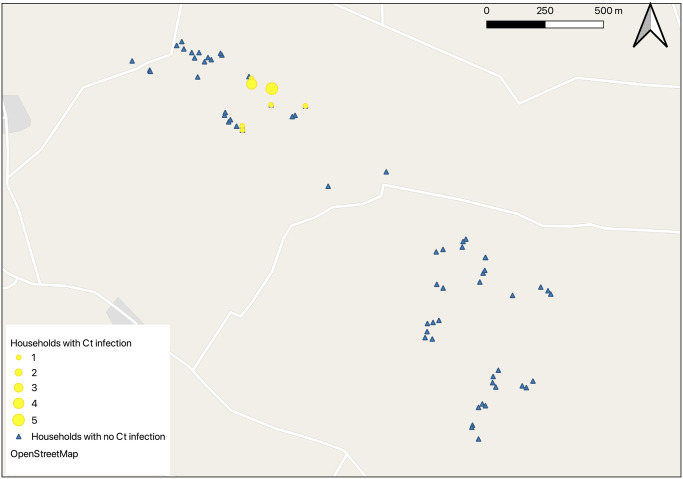
Map of the study area showing a clustered distribution of conjunctival Chlamydia trachomatis (*Ct*) at visit one. Note: Each point shows a household. Geographical maps were created using QGIS and Map base layers taken from OpenStreetMap https://www.openstreetmap.org/copyright generated in QGIS.

The median number of household residents was 4, with an interquartile range of 3–6 and a total range of 2–10. The geographical distribution of the households is illustrated in Figs 1 and 2. Latrines were found in 37/68 (54.4%) of households, and of these 34/37 (91.8%) had evidence of use. Nearly half (48.0%) of the households resided in single-room houses.

### Clinical observations

The prevalence of clinical signs for visit 1 is shown in Table 1. At visit 1 TF was observed in 27/299 (9.0%) all-ages, and in 27/133 (20.0%) of 2–9 year olds. At visit 2 TF was observed in 39/256 (15.0%) all-ages, and in 37/126 (29.4%) of 2–9 year olds. At visit 1 TI was observed in 8/298 (2.7%) all-ages, and in 8/133 (6.0%) of 2–9 year olds. At visit 2 TI was observed in 18/257 (7.0%) all-ages, and in 15/126 (11.9%) of 2–9 year olds.

Signs of active trachoma were identified in one or more individuals in 20/68 (29.4%) households at visit one and 27/68 (39.7%) households at visit two. Within the study village, there was a moderate indication of geographical clustering for both TF or TI, as demonstrated by the Intra-cluster correlation (ICC) values of rho = 0.405 for TF (Fig 1) and rho = 0.386 for TI.

Among the seven individuals initially categorized as having mild disease (F1 and or P1/P2) at visit one, six (85.7%) had developed more severe disease (F2/F3 and or P3) by visit two. Among the 46 people identified as having F2, F3 and/or P3 during visit one, 25/46 (54.3%) maintained this severity, 21/46 (45.7%) showed a reduction in severity to F1 and/or P1/P2, and 7 people who were graded F0P0 at visit one had developed disease signs of F2/F3 and/or P3 by visit 2. No individuals showed clearance of active trachoma signs between the two visits.

During visit one, the prevalence of ocular discharge among children aged 2 to 9 years was 24/133 (18.0%), while nasal discharge was observed in 38/133 children (28.6%), Table 1. At visit two ocular discharge was observed in 7/126 children (5.6%), and nasal discharge was present in 25/126 children (19.8%). There was a significant reduction in ocular discharge from visit one to visit two (χ^2^ test, p = 0.003). There was no difference in nasal discharge between the two visits (χ^2^ test, p = 0.269). Flies were observed on the faces of 62/298 (20.8%) people during the examination at visit one. Among these, 50/62 (80.6%) were children aged 2 to 9 years old. At visit two, flies were observed on 29/257 (11.3%) participants, with 27/29 (93.1%) being children aged 2 to 9 years old.

### Detection of ocular *Chlamydia trachomatis*

Ocular *Ct* infection was detected in 16/298 (5.4%) and 16/257 (6.2%) at visits one and two, respectively. Most infections were detected in children. For children aged 2–9 years infection was detected in 14/133 (10.5%) at visit one and 13/126 (10.3%) at visit two (Table 2). Among those who had been ocular *Ct* positive at visit one, by visit two, four became *Ct* negative, 12 remained *Ct* positive. In addition, four individuals had become ocular *Ct* positive at visit 2.

**Table 2:** Detection of *Ct* DNA and RNA, by clinical status and location at the two visits for children aged 9 years and below.

Variable	Categories	Visit one						Visit two					
		DNA detected			RNA detected			DNA detected			RNA detected		
		n/N^+^	(%)	P-value	n/N^+^	%	P-value	n/ N	%	P-value	n/ N	%	P-value
**Detection in conjunctiva**													
TF	No	4/106	(3.8)	0.002	3/4	75.0	0.308	1/89	1.1		1/1	100.0	1.000
	Yes	10/27	(37.0)		9/10	90.0		12/37	32.4	<0.001	10/12	83.3	
Follicles	F0	3/95	3.2	0.076	2/3	66.7	0.462	1/77	1.3	<0.001	1/1	100.0	0.333
	F1	1/11	9.1		1/1	100.0		0/12	0.0		0	0.0	
	F2	2/10	20.0		2/2	100.0		4/15	26.7		2/4	50.0	
	F3	8/17	47.1		7/8	87.5		8/22	36.4		8/8	100.0	
TI	No	8/125	6.4	0.060	6/8	75.0	1.000	8/111	7.2	0.085	4/8	50.0	0.417
	Yes	6/8	75.0		6/6	100		8/15	53.3		7/8	87.5	
Papillary	P0	2/95	2.1	0.036	1/4	25.0	0.231	0/53	0.0	0.386	0/4	0	0.417
	P1	4/17	23.5		4/4	100.0		1/25	4.0		1/1	100	
	P2	2/13	15.4		1/2	50.0		5/33	15.2		3/5	60.0	
	P3	6/8	75.0		6/6	100.0		7/15	53.3		7/7	100.0	
**Detection on skin**													
Faces		14/105(MV = 28)	13.33		6/14	42.9		13/122(MV = 11)	10.7		----	---	
Hands		11/105(MV = 28)	10.5		4/11	36.4		15/122(MV = 11)	12.3		----	---	

Notes:

1. Denominators for RNA were taken for only DNA positive samples.

2. A sample is considered DNA positive if it is positive for either omcB or plasmids.

3. p-values from logistic regression, except in cases where a cell has fewer than 5 observations, in which case Fishers exact test used.MV–missing values.

4. Viability test was not conducted for faces and fingers at visit two

**Fig 3:**
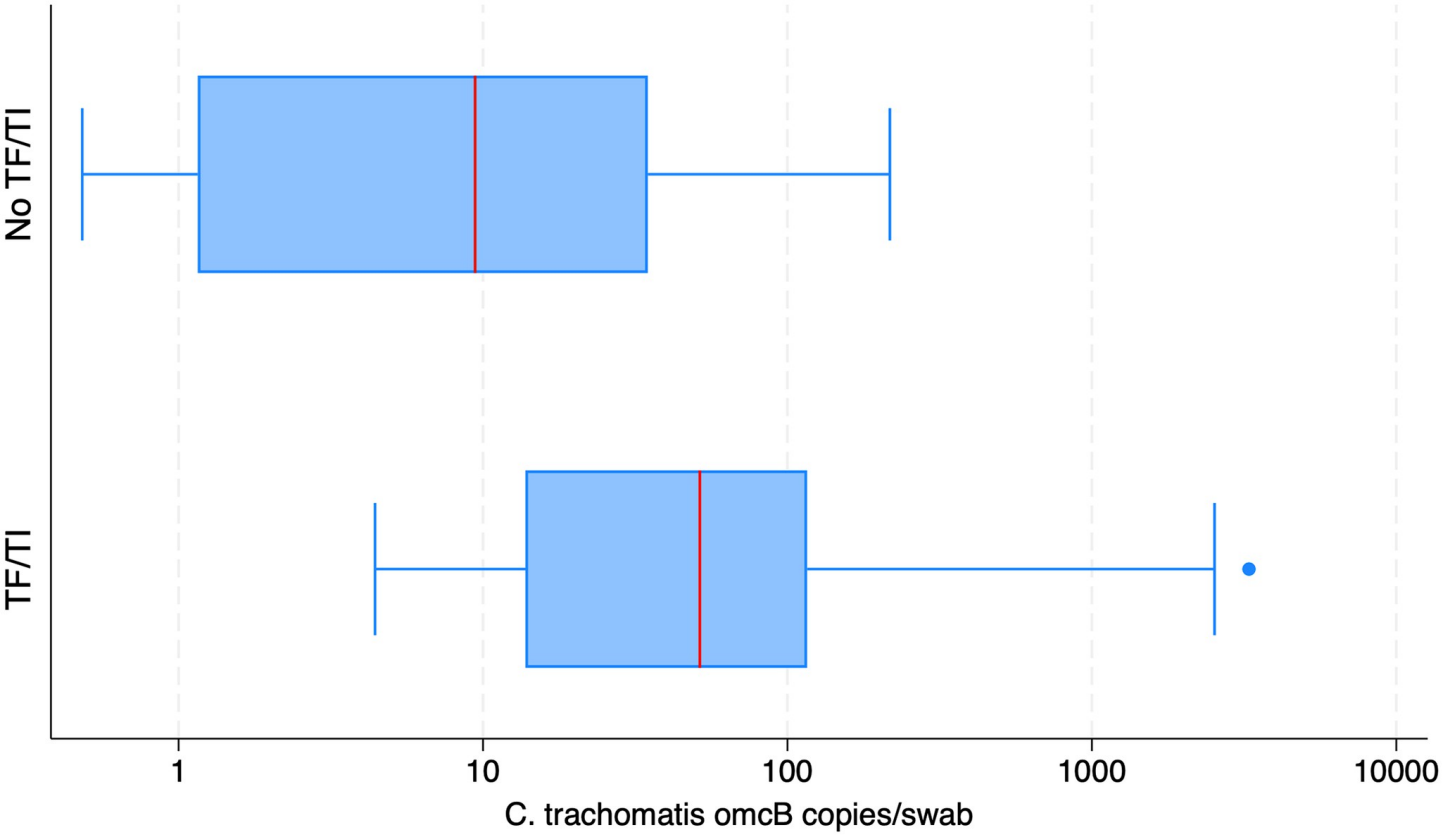
Box-and-whisker plot showing the median *C*. *trachomatis* load (omcB copies/swab) among individuals with or without active trachoma (TF and or TI) during examination at both visits.

*Ct* was detected with increasing frequency with increasing severity of F and P scores (Table 2). *Ct* detection was strongly associated with TF (P = 0.002) and TI (P = 0.060), at visit one (Table 2). High load *Ct* omcB was associated with TI only at visit two (χ^2^ test, p = 0.015). The median ocular *Ct* (omcB) load for children aged 2–9 years old was 48 copies/μl at visit one. At visit two, this was lower at 24 copies/μl (Fig 3).

Ocular *Ct* infection was highly clustered in the same area of the village at both time points, being concentrated in eight households at visit one (ICC, rho = 0.69, Fig 2) and in nine at visit two where 8/9 were the same households that had infection at visit one.

### Detection of *Chlamydia trachomatis* on hands, faces and the association with ocular detection

Across the two study timepoints, we collected and analysed a total of 506 face swabs (252 during visit one and 254 during visit two) and 505 hand swabs (251 during visit one and 254 during visit two). During visit one, samples were obtained from 252 out of 298 individuals (84.6%), and during visit two, samples were obtained from 254 out of 257 individuals (98.8%).

At visit one, of 252 individuals sampled, 16 (6.3%) had detectable *Ct* on their face, among whom four individuals (aged 4, 6, 32, and 35 years) did not have detectable ocular *Ct*. Notably, all except one of these individuals were from households with at least one child had current ocular *Ct* detected. The individuals aged 32 and 35 years are caregivers in households with four and five children with detectable ocular *Ct* infections. Among children (2–9 years), *Ct* was detected on faces at visit 1 in 14/105(13.3%).

**Fig 4:**
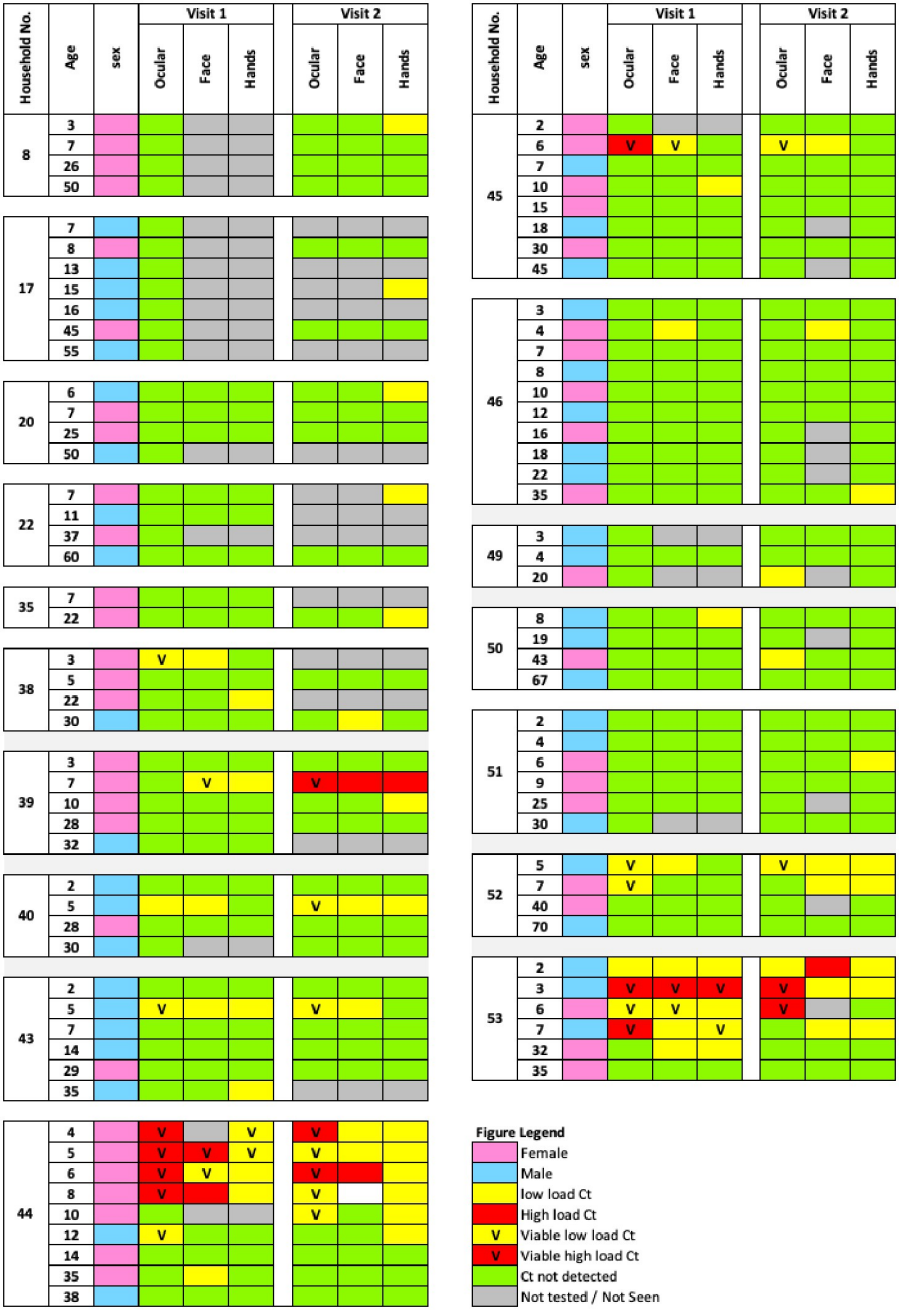
Household distribution of Chlamydia trachomatis in ocular, hand and face samples at visits one and two. **Note**: Each divided block shows households and each row represents an individual with their age and *Ct* infection status. The high load was defined as the geometric mean load exceeding 29 copies/μl across the two combined visits. Swab samples described as "not tested" were not collected. Samples were considered viable if omp2 cDNA amplified with 40 cycles(details in the methods section). Viable samples with high load were defined as “viable high load Ct”, and viable samples with low load were defined as “viable low load Ct”.

Similarly, at visit two, of the 254 individuals sampled, 14 (5.5%) had detectable *Ct* on their face, among which two individuals aged 4 and 30 years tested negative for ocular infection. Interestingly, there was no ocular infection in the 4-year-old’s household. Upon examining their family kinship, both the 4 year old and the 30 year old individuals were cousins residing in neighbouring households with *Ct* infection (Fig 4). The prevalence of *Ct* detection on faces for children aged 2–9 was 13/122 (10.7%).

At visit one, 15/251 (6.0%) hand swabs had detectable *Ct*, among which six individuals aged 6 to 35 had no detectable ocular *Ct*. Notably, none of the six individuals originated from a household with ocular *Ct* infection, but all of them had relatives living in neighbouring households with individuals infected with ocular *Ct*. The prevalence of *Ct* detection from hands for children aged 2–9 was 11/105 (10.5%).

At the second visit, 21/254 (8.3%) hand swabs had detectable *Ct*, among which 10 individuals aged 3 to 40 had no detectable ocular *Ct* infection. Five out of these 10 individuals were immediate neighbours to households with ocular *Ct* infection. The prevalence of *Ct* on hands for children aged 2–9 was 15/122 (12.3%).

### Detection of viable *Chlamydia trachomatis*

Among individuals (all ages) with detectable ocular *Ct* DNA, 13/16 (81.3%) and 12/16 (75.0%) had viable ocular *Ct* at visits 1 and 2, respectively. Among children aged 2–9 years, 12/13 (92.3%) and 11/12(91.7%) had viable ocular *Ct* at visit one and visit two, respectively.

Among children aged 2–9 years, 6/14 (42.8%) had viable *Ct* on their faces at visit one (Table 2). Similarly, in for 2–9 year olds with detectable *Ct* DNA on hand swabs, 4/11 (54.5%) had viable *Ct* on their hands (Table 2).

The air swabs were all negative for the presence of Ct, with only one air swab being a low-level positive for RPP30, which was deemed acceptable.

## Discussion

Here, we present two consecutive assessments of trachoma distribution, two weeks apart in a rural community in Ethiopia. A cohort study offers the opportunity to study disease dynamics in a way that cross-sectional programmatic trachoma surveillance cannot. We observed that 86% of children initially diagnosed with mild disease progressed to more severe disease by visit two. Conversely, among children initially classified with severe disease, 54% maintained their severity level, while 46% showed a reduction in severity. 75% of children with ocular *Ct* infection continued to exhibit the same status two weeks later. Investigating this phenomenon over a longer period within this community would be of interest. Consistent with earlier studies, both the disease and *Ct* infection were found to be primarily confined to a minority of households with limited movement of infection from infected households to uninfected households [28,29].

These results are also consistent with an earlier study we conducted in West Arsi, Oromia region, Ethiopia, indicating that individuals with ocular *Ct* infection also had *Ct* detected on their faces and hands [8]. A proportion of ocular *Ct* positive cases were viable (86.7% at visit one and 75.0% at visit two) for all ages. This is comparable to the rates of viability in studies of urogenital Ct, which ranged from 67–89%, depending on sample type, in a recent meta-analysis [30]. In contrast, despite the difference in the target detected, an earlier study we conducted in the Gambia found only 34.7% of *Ct* DNA positive swabs had detectable *Ct* 16s rRNA, an alternative hypothesised marker for metabolic activity [14]. Our present study extends this investigation further by examining the viability of *Ct* on faces and hands. The viability was found to be highest in eye swab samples, followed by faces and then hands. This observation might be anticipated as the conjunctiva is a mucosal surface that supports active replication. The exposure of *Ct* on hands and faces to direct UV light and a desiccating environment, may cause a decline in its viability. A laboratory-based study from our group, using a dye exclusion assay (PMA) for viability, demonstrated that there was a faster decline in detectable load on spiked pig skin, compared to spiked swabs or plastic surfaces, underscoring this difficulty [10]. *Ct* detected on faces and hands were found to be viable, indicating that these surfaces could serve as routes of transmission. A potential example of this is the case of a 7-year-old girl (from household 39): during the initial visit, detectable *Ct* was limited to her hands and face. However, two weeks later, a viable ocular infection with a high load of *Ct* was detected, along with a high load of *Ct* on both face and hand swabs. Furthermore, *Ct* infection was detected on another individual’s hand within the same household at two weeks. It is noteworthy that this household had immediate neighbours with viable ocular infections.

These observations add to our understanding of potential *Ct* transmission routes. Of relevance is the behaviour of children, who may frequently touch surfaces and the hands of others, transferring contaminants from other children’s hands or faces to their eyes. We also detected *Ct* infections from two individuals living in separate households who had high-load viable ocular infections, along with *Ct* detected on their faces at both time points. However, in these households, no other individuals showed evidence of infection. It is challenging to elucidate why these individuals are not transmitting the infection to their younger siblings, considering similar risk factors. Although not viable, the identification of *Ct* in samples from parents (two from the mother’s hands and one from the father’s face) highlights the potential role of caregivers in transmission.

Children’s faces, particularly ocular discharge at visit two, appeared cleaner compared to the first visit. This observed change may be attributed to parents, having become aware of the facial cleanliness examination after the initial visit, pre-emptively cleaned their children’s faces before presenting them for examination during the second visit. The difference in the decrease in nasal discharge was not significant, while there was a significant increase in active trachoma (TF/TI) compared to the first time point.

This study has several limitations. First, it was not possible to include children under two years in our study, which may be an important group for infection transmission. Second, a number of individuals were not examined at both timepoints. While there were 113 children examined at both timepoints, there were 13 children examined at Visit 1 (of whom 2 had viable ocular Ct infection) but not Visit 2. There were also eight children (none of whom had ocular Ct infection) examined at Visit 2 but not Visit 1. The study area’s vulnerability to drought is a significant factor, leading adults to commute to the regional town each day, instead of participating in farming activities. This travelling poses challenges in consistently observing people. Nevertheless, efforts were made to mitigate this limitation by conducting fieldwork early in the morning before people left the area. Third, there is little empirical data evaluating how mRNA detection or other viability methods relate to true viability of organism in the context of Ct. There is good evidence that omp2 mRNA is detected in live, cultured organism and the signal is drastically reduced or lost completely upon antibiotic or heat treatment [16,27]. However, to further validate the viability of the detected bacteria, future studies incorporating bacterial culture methods would be beneficial, as this could provide more convincing evidence of actual bacterial viability. Fourth, we were unable to provide kappa statistics for ocular and facial secretions between the nurse and the trainer with the assumption that our nurses are well-trained in Stronger SAFE clinical trial standard operating procedure.

## Conclusions

In this dataset, we described the hygiene and sanitation situation of the study community- and systematically described clinical signs and distribution of *Chlamydia trachomatis* from the eyes, faces, and hands through a cross-sectional approach involving two time points. We have used an assay to demonstrate the viability of *Ct* targeting mRNA in Ethiopia. This has provided new insights, demonstrating a proportion of extra-ocular samples to be viable, indicating their potential importance in transmission.

In conclusion, *Ct* needs to remain viable between people if it is going to be transmitted. The detection of viable ocular *Ct* might represent a current ongoing infection rather than just the presence of a remnant of DNA from dead bacteria. Taking this baseline data as a proof of concept, it would be important to systematically investigate how viability changes over a longer period of the cohort study to try to map the transmission and evaluate the impact of interventions (AFE) on infection load and viability.
